# Removal of hazardous textile dye from simulated wastewater by municipal organic solid waste charcoal using machine learning approaches: Kinetics, isotherm, and thermodynamics

**DOI:** 10.1016/j.heliyon.2023.e18856

**Published:** 2023-08-06

**Authors:** Tapos Kumar Chakraborty, Snigdha Ghosh, Md Shahnul Islam, Md Simoon Nice, Khandakar Rashedul Islam, Baytune Nahar Netema, Md Sozibur Rahman, Ahsan Habib, Samina Zaman, Gopal Chandra Ghosh, Md Ripon Hossain, Khadiza Tul-Coubra, Keya Adhikary, Asadullah Munna, Md Muhaiminul Haque, Himel Bosu, Monishanker Halder

**Affiliations:** a Department of Environmental Science and Technology, Jashore University of Science and Technology, Jashore, 7408, Bangladesh; b Department of Computer Science and Engineering, Jashore University of Science and Technology, Jashore, 7408, Bangladesh

**Keywords:** Adsorption, Machine learning approaches, Municipal organic solid waste, Methyl orange dye

## Abstract

This study focuses on the probable use of municipal organic solid waste charcoal (MOSWC) as an adsorbent for Methyl orange (MO) adsorption. The prepared MOSWC is characterized by FE-SEM and FT-IR. Batch adsorption experiments were conducted with the influencing of different operational conditions namely time of contact (1–180 min), adsorbate concentration (60–140 mg/L), adsorbent dose (1–5 g/L), pH (3–11), and temperature (25–60 °C). The high coefficient value (*R*^*2*^ = 0.96) of the process optimization model suggests that this model was significant, where pH and adsorbent dose expressively stimulus adsorption efficiency including 40.11 mg/g at pH (3), MO concentration (100 mg/L), and MOSWC dose (1 g/L). Furthermore, the machine learning approaches (ANN and BB-RSM) revealed a good association between the tested and projected values. The highest monolayer adsorption capacity of MO was 90.909 mg/g. Pseudo-second-order was the well-suited kinetics, where Langmuir isotherm could explain better for equilibrium adsorption data. Thermodynamic study shows MO adsorption is favourable, exothermic, and spontaneous. Finally, this study indicates that MOSWC could be a potential candidate for the adsorption of MO from wastewater.

## 1 Introduction

The rapid development of industrialization and urbanization led to environmental pollution by discharging plentiful toxic chemicals into the environment which is considered as an alarming issue in the globe, especially for developing countries [[1], [2], [3]]. Dyes are a coloured ingredient and considered as most hazardous toxic chemicals used widely in various industries such as textiles, leather, paint, cosmetics, rubber, paper, ceramics, varnishes, pulp mills, ink, plastics, pharmaceuticals, and tanneries [4]. Globally, ten thousand textile dyes are available where over 7 × 10^5^ tones are manufactured yearly and 75% of it is used in the textile industry [5], and more than 50% used dyes are released as coloured effluent during industrial manufacturing and operation steps [5,6]. These effluents are highly toxic for both aquatic and living being [7]. Coloured effluents altered the aquatic ecosystem stability by hindering light penetration, and bleaching, reducing photosynthesis activities and dissolved oxygen, inhibiting fauna growth rate, producing micro toxins, and rising metal chelating [8,9]. Furthermore, exposure to high levels of toxic dyes and their degraded byproducts can create several health hazards to humans such as skin irritation, eye burns, damaging to the kidney, liver, and central nervous system [10]. Therefore, it is necessary to use the treatment of coloured wastewater before discharge into the natural environment. Azo dyes are a versatile class of organic dyes and include 60–70% of all dye production [4,9]. Methyl orange (MO), is a mono-azo dye compound extensively utilize in many industrial parts for ease of application, colour brightness, and excellent binding capacity, but its elimination is challenging due to diverse characteristics (e.g. aromatic ring structure, resistance to degradation, azo link and highly stable to heat and light) [11]. MO is a carcinogenic material; additionally, MO could create several non-carcinogenic human health diseases including vomiting, respiratory tract problems, skin infection, and diarrhoea [12]. MO is mutagenic and becomes very toxic for humans after the metabolism of aromatic amine by intestinal microorganisms [[13], [14], [15], [16]]. Therefore, wastewater must be free from toxicants before releasing into environments for marinating environmental quality and developing an eco-friendly industry. There are numerous treatment approaches exist for the elimination of dyes from polluted water, such as physical methods: adsorption, UV treatment, ion exchange, membrane separation, reverse osmosis, coagulation and flocculation; chemical methods: chemical oxidation processes, ozonation, Fenton's reagent, and photolysis; beside aerobic and anaerobic degradation using microorganisms are designated as biological treatment methods [[17], [18], [19], [20], [21], [22]]. Adsorption is the most effective, informal, and low-cost technique for the treatment of textile effluent other methods due to more convenient, lower cost, higher removal efficiency, lower energy input, effectiveness, and adaptability [23,24]. However, activated carbon are produce from locally available biomaterials (eg. diverse woods, natural coal etc.), which are expensive than marketable activated carbon [25]. Consequently, more recent studies have investigated the use of numerous waste biomasses, such as agricultural wastes [26] and sludge [27], to prepare low/no-cost activated carbon. Biochar (thermal conversion of carbon-containing biomass product is a favourable substitute for commercial activated carbon for cost effectiveness, high adsorption area, and good adsorption capability [28]. On the other hand, different functional groups, including carboxylate, carbonyl, and hydroxyl groups modified bio-char surface charge to eliminate diverse pollutants from polluted water [29,30]. Several studies have been performed to remove pollutants and dyes from aquatic environments, including date seed ash, activated fly ash, coal fly ash, fly ash, zeolite, activated biochar derived from waste materials and burning fly ash [31]. In the experimental study, process modelling and optimization are very important to improve the system performance but conventional methods could optimise a single parameter at a time which increases experimental time and cost [32]. Recently, many researchers used artificial neural networks (ANN) and Response surface methodology (RSM) for optimizing and modelling the diverse experimental parameters at a time which enhances the system performance [33]. Those machine learning approaches are a reliable and powerful tool which helps to overwhelm the system limitations and to assess actual results using experimental data. It is a soft computing technique where required results could be achieved via alternating network weights [32]. So, it does not need any particular understanding of the physical/chemical procedure that moves the system. Nowadays ANN-RSM-based approaches have been used for the diverse area of environmental engineering [[32], [33], [34]]. Literature review indicates that most of the study uses single waste-derived activated carbon (AC) [35] but municipal waste (MW) consists of diverse waste materials, and the AC produced from MW has a larger surface area and comparatively high adsorption performance than other waste materials [36]. Therefore this present study tried to develop activated carbon from municipal organic solid waste using a simple and low-cost process. The ultimate objectives of this study were (i) to investigate the removal performance of MOSWC for MO dye from wastewater using diverse operational conditions such as pH, time of contact, MO concentration, temperature, and MOSWC dose via ANN-RSM modelling; (ii) to explore the adsorption mechanism using diverse models namely, isotherm, kinetic, and thermodynamic; (iii) to assess the further contamination tendency of MOSWC using desorption experiments.

## 2 Materials and methods

### 2.1 Materials and reagents

In the entire experiment, all chemical and reagent was laboratory-grade, purchased from Sigma-Aldrich (Germany), namely MO (≥85%), NaOH (≥97%), and HNO_3_ (69%).

### 2.2 MOSWC preparation and characterization

Municipal organic waste (biodegradable waste) was collected from different households in Jashore, Bangladesh. Firstly, to ensure good mixing, waste was washed with deionized water to remove visible impurities, cut into small sizes, and dried at 80 °C in an electric oven (Oven DSO-500D, Taiwan) until getting moisture free, and cool at ambient conditions. After that, activated carbon was produced by burning in an electric furnace at 500 °C for 30 min retention time in an oxygen-free environment. The burring temperature was selected in previous literature [37,38]. Then carbonize products were crushed and preferred size portions (0.5–1.0 mm) were collected through a conventional sieve. Finally, store it in an air-tight glass bottle for the next experiments. Synthesize MOSWC was characterized using FT-IR, and FE-SEM. The surface morphology of prepared MOSWC was investigated with FE-SEM, Zeiss Sigma 300, Carl Zeiss, Germany, at 10 kV voltages. Before analysis, the MOSWC powder was coated with gold for better imaging and to escape the addition of native electrical charges. The surface chemistry was investigated by FTIR (Nicolet™ iS20, Thermo Scientific, USA), where the recorded spectra range varied from 400–4000 cm-1 with 50 scans attained at 4 cm-1 resolution.

### 2.3 Adsorption experiments

The required quantity of dye powders are dissolved in distilled water for preparing 1000 ppm stock solution and kept the stock solutions pH was less than 3.0 using HNO_3_, then successive dilution approaches were used to prepare the preferred working solution from the stock solution. Adsorption of MO dye onto MOSWC was carried out in a batch mode at 200 rpm using the following operational conditions: pH (3–11), time of contact (1–180 min), temperature (25–60 °C), MOSWC dose (1–5 g/L), and MO concentration (60–140 mg/L). For pH adjustment, 0.1 N acid (HNO_3_) and base (NaOH) solution was used. After a certain time, samples were taken, and, filtered for MO concentration analysis using a UV–visible spectrophotometer (HACH DR 3900, USA) at 464 nm wavelength. A duplicate test was conducted to gating accurate results. The MO adsorption rate and removal efficiency was estimated using Eq. (1) and Eq. (2), respectively.

(1) $$q_{e}=\frac{\left(C_{0}-C_{e}\right)V}{m_{s}}$$

(2) $$R\;\left(\%\right)=\frac{\left(C_{0}-C_{e}\right)}{C_{0}}\times 100$$

where,

C_o_ = initial MO concentration (mg/L)

C_e_ = equilibrium MO concentrations (mg/L),

q_e_ = amount of MO adsorbed (mg/g)

V = volume of liquid solution (L)

m_s_ = MOSWC mass (g).

### 2.4 Adsorption isotherm and kinetic experiments

Adsorption isotherm studies were carried out into 250 mL MO dye solutions of varied MO dye concentrations (60–140 mg/L), at pH 3, where 1 g/L MOSWC dose was added and stirred the solution at 200 rpm with ambient temperature for 90 min. While kinetics experiments were run in 300 mL MO solution at the fixed concentration (60 mg/L) and kept the other condition constant, then samples were taken out after following time intervals 1, 5, 7, 10, 15, 20, 30, 60, 90, 120, 150, and 180 min, filtered, and analyzed. This study applied Langmuir and Freundlich for equilibrium data modelling while pseudo-first-order and pseudo-second-order were used for kinetic modelling, detailed presented in Table S1.

### 2.5 Error analysis

Error analysis is applied for every used model to assess the level of error as compared with obtained and experimental results. In this study, residual sum square (RSS) chi-square (χ^2^) tests, and root means square errors (RMSE) error analysis test (Eqs. (3), (4), (5))) were calculated to determine which adsorption isotherm and kinetic models fitted to experimental data. A smaller error value denotes the model that fits the data the best. Eq. (3) – Eq. (5) describe the formula for determining the best-fit model.

(3) $$RSS=\sum {\left(q_{\exp}-q_{cal}\right)}^{2}$$

(4) $$\chi 2=\sum \frac{{\left(q_{\exp}-q_{cal}\right)}^{2}}{q_{cal}}$$

(5) $$RMSE=\sqrt{\frac{\sum_{i=1}^{n}{\left(q_{\exp}-q_{cal}\right)}^{2}}{n}}$$

where q_exp_ is the observed experimental adsorption data (mg/g) from the kinetic models, q_cal_ is the calculated adsorption data (mg/g) from models, and n represents the number of data sets.

### 2.6 Desorption study

For the desorption study, MO-loaded MOWSC was attained from adsorption isotherm experiments, filtered, and dried. Finally, the experiment was conducted in distilled water with diverse pH and stirring the solution at 200 rpm for 90 min. Eq. (6) was applied for calculating the outcome.

(6) $$Desorption\;\left(\%\right)=\frac{Mass\;of\;MO\;desorbed\;\left(mg/L\right)}{Mass\;of\;MO\;adsorbed\;\left(mg/L\right)}\times 100$$

### 2.7 Adsorption thermodynamics

Thermodynamics is the most vital parameter for an adsorption study, where temperature variation is needed for conducting this study. In this study, Gibbs free energy change (ΔG), enthalpy (ΔH) and entropy (ΔS) are calculated by applying the following Eqs. (7), (8), (9).

(7) $$K_{d}=\frac{q_{e}}{c_{e}}$$

(8) $$\Delta G=-RT\;\ln\;K_{d}$$

(9) $${lnK}_{d}=\frac{\Delta S}{R}-\frac{\Delta H}{RT}$$

where, T = temperature (K), *K*_d_ = equilibrium constant, R = universal gas constant (J mol^−1^ K^−1^)

### 2.8 Process optimization

Box- Behnken designing (BBD) approach is an appropriate statistical tool widely used for process optimization, where the least number of experiments were applied to explore the probable association between the experimental parameters and their influences on the adsorbate adsorption [26]. This study uses three-level three factorial BBD where three factors are defined as X_1_ = pH, X_2_ = MO concentration, and X_3_ = MOSWC dose and three levels are stated as upper (1), central (0), and lower (−1), detailed presented in Table 1. This model runs 17 experiments using Stat-Ease software (Design-Expert 13.0 trial version, Stat-Ease, Inc.). The following polynomial equation (Eq. 10) is used for BBD modelling.

(10) $$Z=\beta_{0}+\sum_{I=1}^{n}\beta_{i}A_{i}+\sum_{i=n}^{n}\beta_{ij}A_{i}A_{j}+\sum_{i=1}^{n}\beta_{ii}A_{i}^{2}$$

where Z is the projected response of MO adsorption (mg/g);, β_0_ = Constant, β_i_ = linear coefficient. β_ij_ = interface coefficients, β_ii_ = quadratic coefficients, and A_i_ and A_j_ = process variables.

**Table 1:** Independent variables, their experimental range and three levels of these variables.

Factors	Levels		
	−1	0	+1
X_1_: Solution pH	3	5	11
X_2_: MO dye concentration (mg/L)	60	100	140
X_3_: Activated carbon ratio (g/L)	1	3	5

## 3 Results and discussion

### 3.1 Characterization of adsorbent

**Fig. 1:**
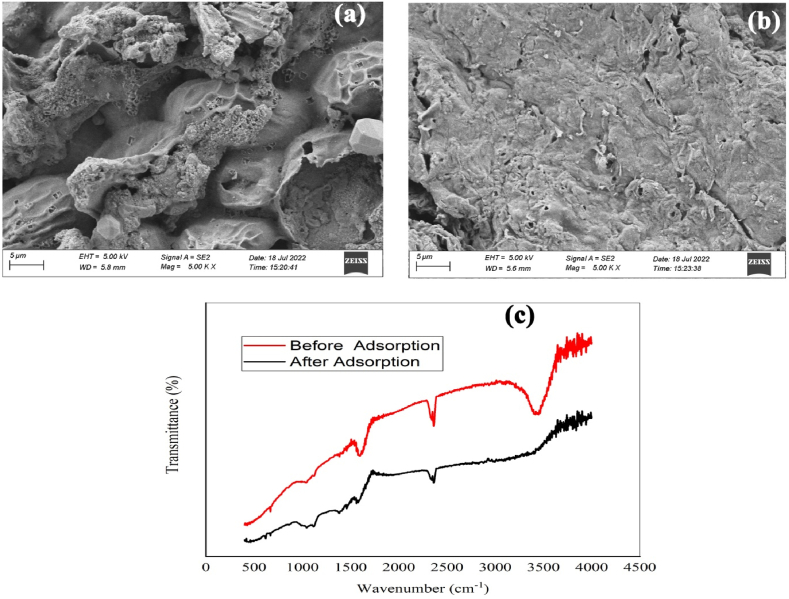
FE-SEM photographs (a. before adsorption; b. after adsorption) and FTIR spectra of MOSWC (c).

Municipal organic solid waste compost of diverse active compounds including lignin, cellulose, pectin, hemicellulose etc which promote to adsorb toxic ions with different functional groups (eg. COOH, –OH, –NH, C=O, C=C, etc) [29]. The surface chemistry of the adsorbent is assessed using FTIR analysis to identify the nature and diversity of this material. The FTIR spectrum for MOSWC (before and after adsorption) shows specific absorption peaks presented in Fig. 1c. The major adsorption peaks were found at 3629-3448 cm^−1^ region indicating O–H stretching and –NH groups. The peaks observed around 1745, 1654, and 1596 cm^−1^ indicated the stretching vibration of C=O, C=C, and –CN, respectively. The aromatic C–H bending vibrations were observed at 668 cm^−1^. Slight alterations were observed after MO adsorption (Fig. 1c), while above mentioned adsorption peaks can link with the MO molecules while more specifically hydroxyl group (OH) and heteroatoms highly regulate the linkage between adsorbent and adsorbate. Similar results were demonstrated by Ghosh et al. [17] and Chakraborty et al. [4]. The surface feature of MOSWC (before and after adsorption) was estimated through SEM analysis, attained results presented in Fig. 1 (a, b). Raw MOSWC exhibits lots of pores and a rough surface (Fig. 1a) while a smooth surface was observed after the adsorption of MO dye (Fig. 1b).

### 3.2 Effects of process variables for MO adsorption

#### 3.2.1 Effect of contact time

**Fig. 2:**
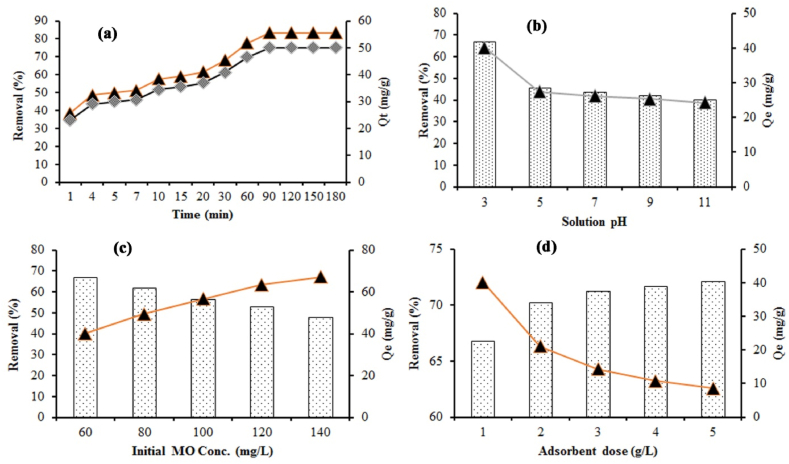
Effects of diverse factors including (a) contact time; (b) pH; (c) initial Mo dye concentration; (d) adsorbent dose for MO adsorption using MOSWC.

To select the equilibrium contact time for MO dye adsorption onto MOSWC, an adsorption experiment was conducted at an initial MO dye concentration of 60 mg/L with the following conditions: pH (3), adsorbent dose (1 g/L), temperature (25 ± 2 °C), 200 rpm rotation speed, after selected time interval (1–180 min) samples were taken from the experimental solution, and results are presented in Fig. 2a. Mainly three stages controlled the whole adsorption procedure: (1) quick adsorption was achieved at an early stage (1–5 min) due to bulk concentration of MO ions and huge vacant space on the adsorbent surface, (2) after 5–90 min the adsorption efficiency are turning into slow due to declining of existing binding sites with time movement, and (3) finally, the adsorption efficiency goes to comparatively very low within 90–180 min due to blocking of almost all vacant space (outer and inner site) on the adsorbent surface. So, adsorption researched equilibrium in 90 min, which was chosen as the equilibrium contact time for further experiments. A similar outcome has been reported for textile dye adsorption using bio-adsorbent [5,6,11].

#### 3.2.2 Effect of pH

For adsorption science, pH is considered a vital factor because it influences dye ionization rate, modification of adsorbent inner and outer binding sites, and shifting of adsorbent surface charge [4]. Fig. 2b shows the adsorption performance of MO dye using MOSWC at varied solution pH 3 to 11. The MO removal efficiency (67–40%) and adsorption rate (40.11–24.11 mg/g) by MOSWC gradually reduce with rising solution pH (pH 3–11), and the maximum result was found at pH 3 (67% and 40.11 mg/g) (Fig. 2b). In an acidic environment, the adsorbent surface becomes positive (R_3_-NH^+^) and in addition to H-bonding enhanced the electrostatic interaction increases between adsorbent and adsorbate, consequence achieved maximum adsorption [39], characterized by the following equations. In an aqueous environment, MO converted into anionic colours after dissociation of the R–SO3^-^ group.

(11) $$R-{SO}_{3}Na+H_{2}O↔{{R-SO}_{3}}^{-}+{Na}^{+}$$

(12) $${{R-SO}_{3}}^{-}+{{R-NH}_{3}}^{+}↔{{R-NH}_{3}}^{+}O_{3}S-R$$

The *pKa* of MO is 3.8, MO shows a negative attitude (dissociation of –SO^3−^) after exceeding the solution pH 3.8 [40]. Therefore, in a basic environment, the electrostatic repulsions are increased between MO ions and adsorbent surface charges with increasing solution pH, which reduce the adsorption performance of MOSWC [6,24].

#### 3.2.3 Effect of initial MO dye concentration

Fig. 2c shows the elimination rate of MO decreases (67%–48%) with rising in initial MO dye concentration ranging from 60 to 140 mg/L because, at a stable dose, the outer layer of the adsorbent is covered by dye molecules. While the adsorption rate of MO gradually rises (40–67 mg/g) with rising MO dye concentration (60–140 mg/L) at constant MOWSC dose (1 g/L) (Fig. 2c), for the higher association between MO molecules and MOSWC that improved the major dynamic potency to shift a high mass of MO between the liquid to the solid phase in an aqueous environment [19,49].

#### 3.2.4 Effect of adsorbent dose

To assess the suitable MOSWC dose, batch adsorption experiments of MO onto MOSWC were conducted at 25 ± 2 °C, with different adsorbent doses (1–5 g/L) and keeping the other condition constant (pH = 3, MO concentration = 60 mg/L). The performance of MO elimination onto MOSWC improved from 66% to 73% with rising MOSWC doses (1–5 g/L) (Fig. 2d), respectively due to more vigorous adsorption sites on the adsorbent surface, while the adsorption rate gradually reduces from 40.11 to 8.65 mg/g with rising adsorption dose might be the overlapping with one another (e.g. assemblage) of MO molecules onto the MOSWC. A related outcome has been stated for the MO dye adsorption using Mahagoni bark charcoal Ghosh et al. [17].

### 3.3 Adsorption isotherms

The linkage between adsorbate and adsorbent in an adsorption study is well explained by Adsorption isotherms [7]. In this present study, Langmuir and Freundlich's isotherms were used, which are usually useful in the solid/liquid system, presented in Table 2. Langmuir was the more situated isotherm for MO adsorption onto MOSWC due to its higher correlation coefficient value (R^2^ = 0.998), lower error (RSS = 1.310), and chi-square value (*χ*^*2*^ = 0.022) and (RMSE = 0.670) as compare Freundlich isotherm (R^2^ = 0.988, RSS = 6.640 χ^2^ = 0.111 and RMSE = 1.025) (Table 2).

**Table 2:** Isotherm model parameters for adsorption of MO onto MOSWC.

Isotherm models	Parameters	MOSWC
Langmuir	*q*_max_ (mg/g)	90.909
	*b* (L/mg)	**0.039**
	*R*_*L*_	**0.717–0.202**
	*R*^*2*^	**0.998**
	***RSS***	**1.310**
	*χ*^*2*^	**0.022**
	*RMSE*	**0.570**
Freundlich	*K*_*F*_ (mg/g) (L/mg)^1/n^	**12.285**
	*n*	2.486
	*R*^*2*^	**0.988**
	***RSS***	**6.640**
	*χ*^*2*^	**0.111**
	*RMSE*	**1.025**

reveal that MO molecules produce a single continuous layer with similar dispersal to MOSWC. The monolayer maximum adsorption capacity of MO onto MOSWC was 90.090 mg/g (Table 2). The R_L_ values of MOSWC (0.717–0.202) were between 0 and 1, representing that MO adsorption onto MOSWC was appropriate under the studied experimental conditions. Conversely, the value of adsorption intensity (n) (*n* = 2.487) was higher than 1 and higher *K*_*F*_ = 12.285, demonstrating that the adsorption process was promising for MO adsorption from aqueous solutions using MOSWC (Table 2). The explanation of this equilibrium study also correspondence with other anionic azo dyes adsorption studies [7]. Additionally, the performance of MO dye adsorption using MOSWC is comparable with other adsorbents, presented in Table S2.

### 3.4 Adsorption kinetics

Adsorption kinetics is needed to design an effluent treatment plant unit, where adsorbate could be removed pollutants at a certain rate. In the present study, two commonly uses kinetics models such as Lagergren's pseudo-first-order, and Ho's pseudo-second-order applied for MO dye adsorption onto MOSWC. The model accuracy depends on the high correlation coefficient (R^2^) and lower error values (RSS, chi-square (*χ*^*2*^) and RMSE) of the model, and the applied model parameters are represented in Table 3. The pseudo-second-order kinetic model shows higher R^2^ and lower RSS, *χ*^*2*^, and RMSE values for Mo dye adsorption as compared with pseudo-first-order kinetic, additionally, the calculated (q_e,cal_) (q_e,cal_ = 51.546) value from the pseudo-second-order kinetic model also corresponding to the experimental value (q_e,exp_) (q_e,exp_ = 49.972 mg/g), showing that pseudo-second-order was best fitted kinetic model for adsorption data. Therefore, chemical adsorption controlled the total adsorption process.

**Table 3:** Kinetic model parameters for MO adsorption onto MOSWC.

Models	Parameters	MOSWC
	*q*_*e,exp*_ (mg/g)	49.984
Pseudo-first order	*q*_*e,cal*_ (mg/g)	24.871
	*K*_*1*_ (min^−1^)	0.035
	*R*^*2*^	0.994
	*RSS*	8287.3
	*χ2*	46.21
	*RMSE*	26.027
Pseudo-second order	*q*_*e,cal*_ (mg/g)	51.546
	*K*_*2*_ (g/mg min)	0.004
	*H* (mg/g min)	10.660
	*R*^*2*^	0.999
	*RSS*	301.79
	*χ2*	0.61
	*RMSE*	5.014
Intraparticle diffusion	*K*_*diff*_ (mg/gmin^0.5^)	2.670
	*C* (mg/g)	24.144
	*R*^*2*^	0.951

**Fig. 3:**
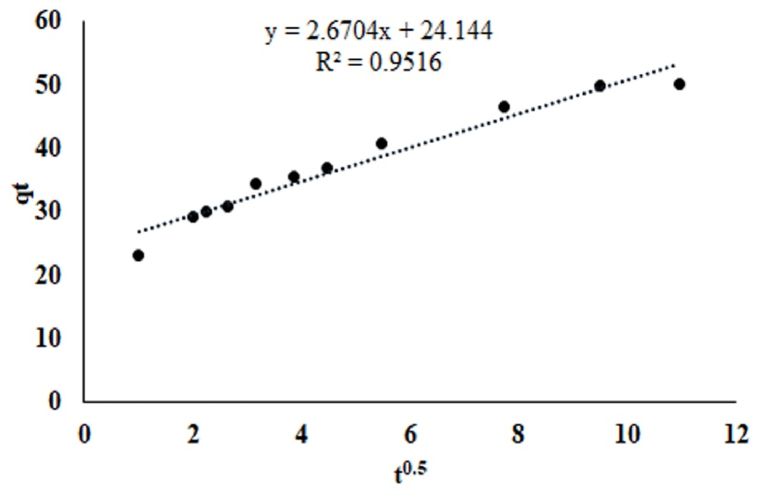
Intra-particle diffusion plot for MO adsorption onto MOSWC.

The experimental data were also evaluated by intraparticle (IP) diffusion to determine the diffusion mechanisms. The IP plot did not pass through the origin (Fig. 3) and high intercept values (*C* = 24.144) (Table 3), representing that exterior diffusion was the rate-limiting step as interior diffusion for MO dye adsorption using MOSWC, which may happen at the same time.

### 3.5 Optimization of process variables

#### 3.5.1 Box–Behnken design and regression model

**Fig. 4:**
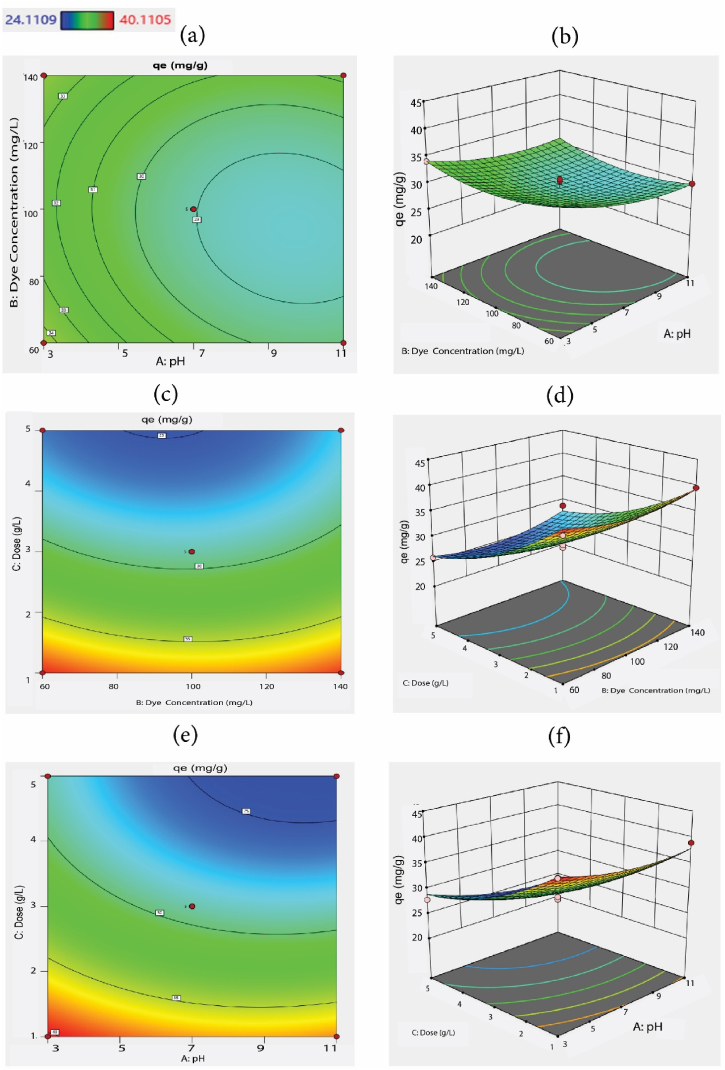
BBD 2D and 3D response plots for MO adsorption using MOSWC: (a, b) solution pH; (c, d) initial MO concentration, and (e, f) adsorbent dose].

For process optimization, the three factors including pH, initial MO dye concentration and the adsorbent dose were applied and the studied responses are presented in Table S3 and Fig. 4, respectively.

The adsorption capacity for MO ranged between 24.11 and 40.11 mg/g (Table S3). The highest result was found for 40.12 mg/g at an acidic medium (pH 3) with a 1 g/L adsorbent dose (Fig. 4). The study result shows the H^+^ concentration on the adsorbent surface is increased in an acidic environment, which enhanced the interaction between adsorbate-adsorbent, consequently, the maximum result was achieved at low pH (3) [41]. The association and fitness between response and experimental data were validated using regression analysis, where the role of particular variables and their interface, are presented in Fig. 4b-d. The major association was found between solution pH and adsorbent dose with a positive effect (*X*13 = +0.43), while a negative association existed between solution pH and dye concentration (*X*12 = −0.64) for MO adsorption using MOSWC (Table S4). The statistical link between the nominated experimental factors and the response was explained by a quadratic model with corresponding coded factors and their best fitted using the following Eq. (10).

(13) $$q_{e}\left(MO;mg/g\right)=29.04-1.88X_{1}+0.26X_{2}-6.35X_{3}+0.43X_{1}X_{2}-0.65X_{1}X_{3}+0.46X_{2}X_{3}+1.47X_{1}^{2}+1.89X_{2}^{2}+2.23X_{3}^{2}$$

Analysis of variance (ANOVA) is essential for determining the significance of the second-order model and the result of ANOVA for the adsorption of MO is presented in Table 4, where pH and adsorbent dose show a significant effect for MO removal from aqueous solution. Table 4 shows that the second-order model was statistically significant (P < 0.0004).

**Table 4:** Analysis of variance of the fitted second-order model for MO dye adsorption.

Source	Sum of Squares	df	Mean Square	F-value	p-value*	
Model	405.11	9	45.01	18.53	0.0004	significant
*X*_*1*_, pH	28.23	1	28.23	11.62	0.0113	
*X*_*2*_*,* MO dye conc.	0.5286	1	0.5286	0.2176	0.6550	
*X*_*3*_*,* Adsorbent dose	322.78	1	322.78	132.86	<0.0001	
*X*_*1*_*X*_*2*_	0.7396	1	0.7396	0.3044	0.5983	
*X*_*1*_*X*_*3*_	1.67	1	1.67	0.6884	0.4341	
*X*_*2*_*X*_*3*_	0.8557	1	0.8557	0.3522	0.5715	
*X*_*1*_^*2*^	9.14	1	9.14	3.76	0.0536	
*X*_*2*_^*2*^	15.03	1	15.03	6.19	0.0418	
*X*_*3*_^*2*^	21.03	1	21.03	8.65	0.0217	
**Residual**	17.01	7	2.43			
Lack of Fit	9.06	3	3.02	1.52	0.3385	not significant
Pure Error	7.94	4	1.99			
Cor Total	422.12	16				
Adeq Precision	13.77					
*R*^*2*^	0.96					
Adjusted *R*^*2*^	0.91					

*P < 0.05 indicates statistical significance.

**Fig. 5:**
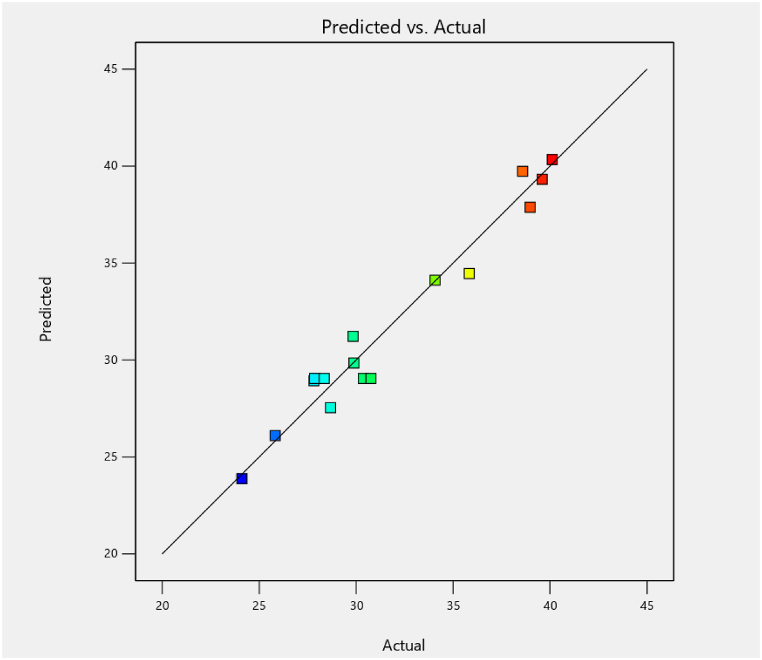
Experimental vs. BBD model predicted MO removal.

The model output shows “Lack of fit” is more significant than pure error. Besides, X_1_, X_3_, X_1_^2^ × _2_^2^, and X_3_^2^, respectively are significant model (p < 0.05) terms for MO adsorption using MOSWC. Adsorbent dose and pH significantly influence MO adsorption. Moreover, the coefficient of determination (*R*^*2*^ = 0.96) showed that 96% of the total variability of the result was explained by this model. A good agreement was attained between experimental (R^2^) and projected (R^2^adj) results for MO (Fig. 5), showing that the model is a good fit, where most of the data points near the straight line. The Model F-value of MO was 18.53 suggesting that the model is significant. There is only a 0.04% chance for MO to create noise due to a higher F-value. The signal-to-noise ratio >4 indicates Adeq Precision. The ratio of signal-to-noise 13.769 shows an adequate signal. Therefore, this model is appropriate for the design. Venkataraghavan et al. [26] and Saha et al. [42] used a related model for the adsorption of textile effluent*.*

#### 3.5.2 Artificial neural network modelling

ANNs are widely used for recording the non-linear relation between independent and dependent variables and are suitable to apply at any condition [32]. This study applies a multi-level feed-forward neural network, which is directed in the following order: input-hidden-output. Applied ANN consists of 60% training, 20% validation, and 20% testing networks. The input parameters (pH, adsorbent dose, and initial MO concentration) were selected for ANN, while the percentage of MO removal was selected as the output layer.

**Fig. 6:**
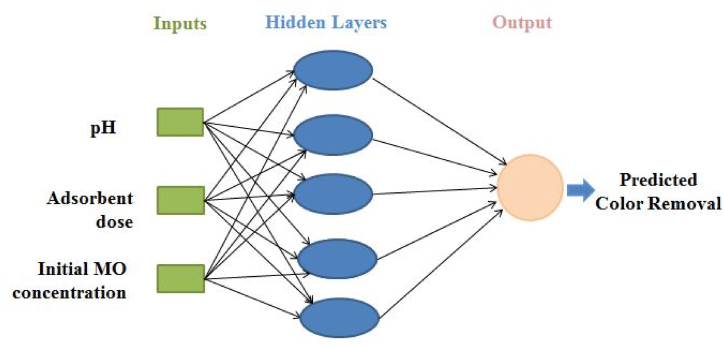
ANN network with topology for MO adsorption using MOSWC.

**Fig. 7:**
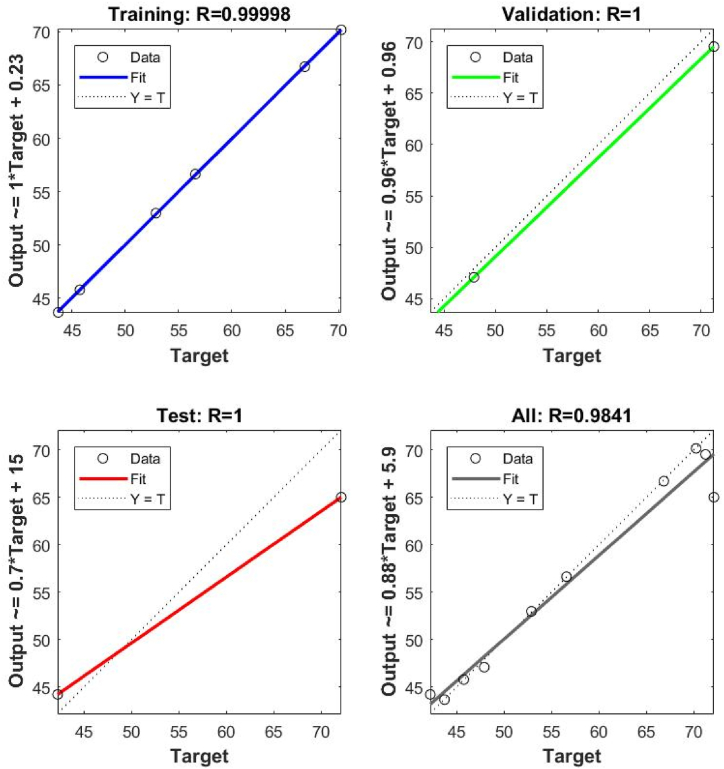
Linear fit for experimental and predicted colour removal using ANN for MO.

The trial and error techniques were applied to achieve the model accuracy and validation and testing are carried out using MATLAB (R2020a). Fig. 6 represents the topology for MO adsorption including 3:5:1 (Table S5). The high and low frequencies of hidden neurons directly affect the ANN presentation and the appraisal of accuracy. So, the ideal quantities of hidden neuron selection assist to escape over and under estimation [32]. ANN performance is improved with rising neuron numbers, but the coefficients of the determinant (R^2^) did not represent the same outcome in the training phase (Table S5). In the case of MO adsorption, all training, validation, and testing phase of tan-sigmoidal and topology was selected according to high R-value and its associated lower MSE value (Table S6). Good associations between experimental and ANN-predicated results (Fig. 7) indicate that the ANN model was suitable for describing MO adsorption using MOWSC. Gadekar and Ahammed [32] and Bhowmik et al. [43] applied similar approaches for dye adsorption modelling using BB-RSM and ANN approaches.

### 3.6 Adsorption thermodynamics studies

**Fig. 8:**
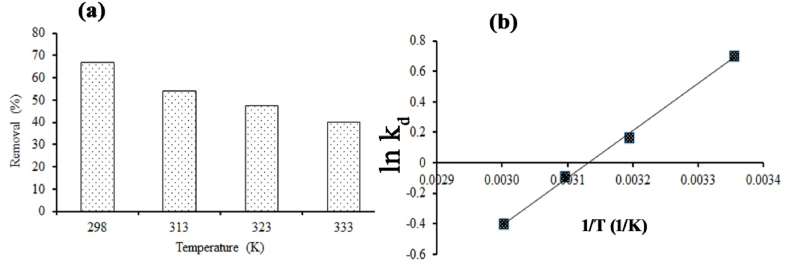
Thermodynamic study of MO removal using MOSWC (a) removal rate and (b) Thermodynamic modelling.

The thermodynamic study represents the role of temperature for adsorption, the nature of the linkage between adsorbate and adsorbent, direction and mechanism of reaction with changing the experimental temperature [53], results are shown in Table 5, while A Van't Hoff plot of lnk_d_ vs 1/T produced a straight line with *R*^*2*^ values of 0.998, shown in Fig. 8b. The negative ΔG^0^ value at 298 K indicates the suitable and practical so, it was selected for further experiments (Fig. 8a) [49] The positive ΔG^0^ values for 323 and 333 K suggest thermodynamically unsuitable, a related result stated by Tay et al. [53].

**Table 5:** Thermodynamic modelling of MOSWC on methyl orange removal.

Temperature (K)	${\Delta G}^{0}$ (J/mol)	${\Delta H}^{0}$ (KJ/mol)	${-\Delta S}^{0}$ (J/mol/K)
298	−1737.89	−25.70	80.51
313	−429.89		
323	248.026		
333	1109.51

The exothermic type of adsorption is confirmed by the negative result of ΔH^0^, MO molecules adsorption decreases with rising temperature, and no remarkable deviations were found after 298 K, supported by Fig. 8a. Therefore, next experiments were conducted at this temperature. The positive ΔS^0^ also suggest that lessening chance between the MOSWC and MO dye molecules interface, which mainly occurred through the chemical adsorption mechanism through the ion exchange process. Chakraborty et al. [54] detect the exothermic reaction for dye adsorption using carbonizes adsorbent.

### 3.7 Desorption study

**Fig. 9:**
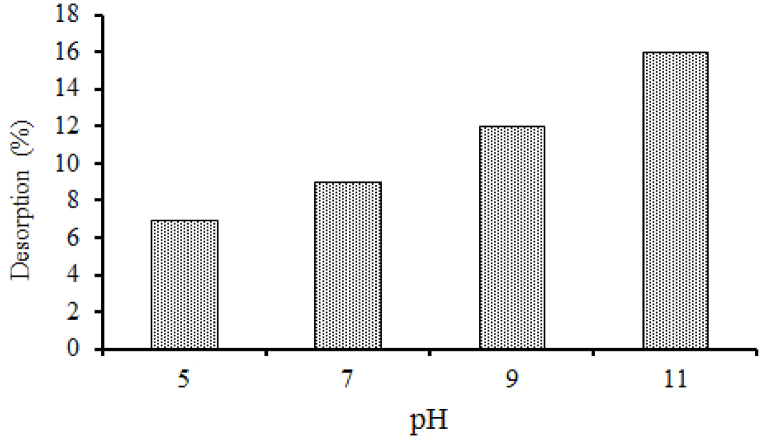
Desorption study of MOSWC at different pH.

Desorption process is applied to assess the possibility of further contamination when the treated adsorbent comes into the environment. This process is wholly controlled by chemical bonding (ionic, covalent, van der Waals' forces or dipole-dipole interaction) between adsorbent and adsorbate [51]. In this study, desorption experiment was conducted with diverse pH values (pH 5–11). Fig. 8 shows the desorption percentage of MOSWC (7–16%) was very low; instead of increasing pH might be the possibility of existing strong chemical bonding between MO molecules and MOSWC (Fig. 9). A Comparable result was also reported by Chakraborty et al. [4] and Çelekli et al. [55].

## 4 Conclusions

This present study explores the usability of MOSWC as a potential biosorbent for MO removal from simulated wastewater under batch adsorption experiments. BBD and ANN models are suitable for MO dye adsorption modelling, where both models showed a good agreement between predicted and experimental results. Quadratic models indicate that solution pH and adsorbent dose significantly influence MO dye removal. Pseudo-second-order was the best-matching kinetic model for MO adsorption data. The equilibrium data well explained by the Langmuir isotherm model with the highest adsorption rate was 90.909 mg/g. Thermodynamic study shows the adsorption is exothermic and spontaneous for MO removal. Cost-effectiveness, obtainability, and favourable study results make the MOSWC a suitable and effective adsorbent for removing dyes and other environmental pollutants. Consequently, MOSWC could be applied for textile effluent treatment, where a centralized wastewater treatment system is not accessible.

## Author contribution statement

Tapos Kumar Chakraborty: Samina Zaman: Conceived and designed the experiments; Analyzed and interpreted the data; Wrote the paper.

Snigdha Ghosh: Conceived and designed the experiments.

Md. Shahnul Islam: Ahsan Habib: Performed the experiments; Wrote the paper.

Md. Simoon Nice: Khandakar Rashedul Islam: Baytune Nahar Netema: Md. Sozibur Rahman: Md Ripon Hossain: Performed the experiments.

Gopal Chandra Ghosh: Conceived and designed the experiments; Analyzed and interpreted the data; Contributed reagents, materials, analysis tools or data.

Khadiza Tul-Coubra: Keya Audhikary: Asadullah Munna: Md. Muhaiminul Haque: Analyzed and interpreted the data.

Himel Bosu: Monisanker Halder: Contributed reagents, materials, analysis tools or data.

## Data availability statement

Data will be made available on request.

## Declaration of competing interest

The authors declare that they have no known competing financial interests or personal relationships that could have appeared to influence the work reported in this paper.
