# Supplementary material for: Removal of hazardous textile dye from simulated wastewater by municipal organic solid waste charcoal using machine learning approaches: Kinetics, isotherm, and thermodynamics
Source: Heliyon. 2023 Aug 6;9(8):e18856. doi: 10.1016/j.heliyon.2023.e18856 (PMC10493414; doi:10.1016/j.heliyon.2023.e18856)
Supplement: Multimedia component 1 [file mmc1.docx]

**Removal of hazardous textile dye from simulated wastewater by municipal organic solid waste charcoal using machine learning approaches: Kinetics, isotherm, and thermodynamic studies**

Tapos Kumar Chakraborty^a^*, Snigdha Ghosh^a^, Md. Shahnul Islam^a^, Md. Simoon Nice^a^, Khandakar Rashedul Islam^a^, Baytune Nahar Netema^a^, Md. Sozibur Rahman^a^, Ahsan Habib^a^, Samina Zaman^a^, Gopal Chandra Ghosh^a^, Md Ripon Hossain^a^, Khadiza Tul-Coubra^a^, Keya Adhikary^a^, Asadullah Munna^a^, Md. Muhaiminul Haque^a^, Himel Bosu^a^, Monishanker Halder^b^

^a^Department of Environmental Science and Technology, Jashore University of Science and Technology, Jashore 7408, Bangladesh

^b^Department of Computer Science and Engineering, Jashore University of Science and Technology, Jashore 7408, Bangladesh

*Corresponding Author: E-mail: [taposchakraborty@just.edu.bd](mailto:taposchakraborty@just.edu.bd)

**Table S1** Adsorption modeling and error analysis procedure

| **Adsorption Model** | **Equation** | **Parameter** | **References** |
| --- | --- | --- | --- |
| Langmuir isotherm | $q_{e}=\frac{q_{\max} b C_{e}}{1+bC_{e}}$ | Ce (mg/L)= equilibrium concentration of dye.  qe (mg/g)= amount of dye adsorbed at equilibrium.  q_max_(mg/g)=maximum monolayer coverage  b (L/mg)=Langmuir constant. | [44] |
|  | $R_{L}=\frac{1}{1+bC_{0}}$ | R_L_= separation factor  unfavourable, linear, favourable, and irreversible R_L_> 1;  R_L_ = 1; 0 < R_L_<1; and R_L_ = 0, respectively. |  |
| Freundlich isotherm | $q_{e}=K_{F}C_{e}^{1/n}$ | K_F_ = Freundlich constant.  n=adsorption intensity. | [45] |
| Pseudo-first-order kinetic | $q_{t}=q_{e} \left( 1-e^{-k_{1}t} \right)$ | K_1_ = pseudo-first-order rate constant (min^-1^), q_e_, and q_t_ are the quantity of dye adsorbed (mg/g) at equilibrium and time, t. | [46] |
| Pseudo-second-order kinetic | $q_{t}=\frac{K_{2}q_{e}^{2}t}{1+K_{2}q_{e}t}$ | K_2_ = pseudo-second-order rate constant (g/mg/min). | [47] |
| Intraparticle diffusion | $q_{t}={K_{diff.}t}^{0.5}+C$ | K_diff_. = intraparticle diffusion rate constant (mg/g min^0.5^),  C =intercept. | [48] |

**Table S2** Comparison of MO dye adsorption capacity by MOSWC with other adsorbents

| Adsorbents | Maximum adsorption capacity (mg/g) | Optimum dose (g/L) | Concentration range (mg/L) | References |
| --- | --- | --- | --- | --- |
| Activated Pomelo peel waste | 163.105 | 1.0 | 30-150 | [11] |
| Activated *Vitis vinifera L.* | 111.11 | 1.0 | 100-1000 | [5] |
| Municipal organic solid waste charcoal (MOSWC) | 90.91 | 1 | 60-140 | This study |
| Coffee waste/cetylpyridinium chloride | 62.5 | 2 | 20-120 | [50] |
| Modified wheat straw | 50.4 | 1 | 30-80 | [51] |
| Mahagoni (*Swietenia mahagoni*) Bark | 6.071 | 10 | 10-100 | [17] |

**Table S3.**  Box–Behnken design matrix for MO dye removal

|  | | **Coded values** | | | **Actual values** | | | **Adsorption** |
| --- | --- | --- | --- | --- | --- | --- | --- | --- |
| **Run** | **X_1_** | | **X_2_** | **X_3_** | **Solution pH** | **Dye Concentration (mg/L)** | **Activated Carbon Ratio (g/L)** | **q_e_ (mg/g)** |
| 1 | | 1 | 1 | 0 | 11 | 140 | 3 | 29.84 |
| 2 | | 0 | 0 | 0 | 7 | 100 | 3 | 30.37 |
| 3 | | 1 | 0 | -1 | 11 | 100 | 1 | 38.97 |
| 4 | | 0 | 0 | 0 | 7 | 100 | 3 | 30.76 |
| 5 | | 0 | -1 | -1 | 7 | 60 | 1 | 38.59 |
| 6 | | -1 | -1 | 0 | 3 | 60 | 3 | 35.83 |
| 7 | | 0 | 1 | 1 | 7 | 140 | 5 | 28.68 |
| 8 | | 0 | 1 | -1 | 7 | 140 | 1 | 39.59 |
| 9 | | -1 | 1 | 0 | 3 | 140 | 3 | 34.07 |
| 10 | | -1 | 0 | 1 | 3 | 100 | 5 | 27.83 |
| 11 | | 1 | 0 | 1 | 11 | 100 | 5 | 24.11 |
| 12 | | 1 | -1 | 0 | 11 | 60 | 3 | 29.89 |
| 13 | | 0 | 0 | 0 | 7 | 100 | 3 | 27.89 |
| 14 | | 0 | 0 | 0 | 7 | 100 | 3 | 28.35 |
| 15 | | 0 | 0 | 0 | 7 | 100 | 3 | 27.88 |
| 16 | | 0 | -1 | 1 | 7 | 60 | 5 | 25.83 |
| 17 | | -1 | 0 | -1 | 3 | 100 | 1 | 40.11 |

**Table S4**. Values of model coefficients of the two responses

| **Main coefficients** | ***qe* (MO)** |
| --- | --- |
| *b_o_* | 29.05 |
| *X1* | -1.88 |
| *X2* | 0.26 |
| *X3* | -6.35 |
| *X12* | 0.43 |
| *X13* | -0.65 |
| *X23* | 0.46 |
| *X11* | 1.47 |
| *X22* | 1.89 |
| *X33* | 2.23 |

**Table S5**. Comparison of MO color removal prediction using tan-sigmoidal and Log-sigmoidal transfer functions

| **Topo-logy** | **Tan-sigmoidal** | | | | | **Log-sigmoidal** | | | | |  |
| --- | --- | --- | --- | --- | --- | --- | --- | --- | --- | --- | --- |
|  | MSE | R | | | | MSE | R | | | |  |
|  |  | Tr | Val | Test | All |  | Tr | Val | Test | All |  |
| 3:2:1 | 0.02 | 0.88 | 1 | 1 | 0.92 | 2.2 | 0.38 | 1 | 1 | 0.45 |  |
| 3:3:1 | 13.15 | 0.97 | 1 | 1 | 0.78 | 20.6 | 0.92 | 1 | 1 | 0.72 |  |
| 3:4:1 | 0.87 | 0.98 | 1 | 1 | 0.97 | 8.63 | 0.98 | 1 | 1 | 0.98 |  |
| 3:5:1 | 0.10 | 0.994 | 1 | 1 | 0.98 | 1.8 | 0.999 | 1 | 1 | 0.98 |  |
| 3:6:1 | 17.84 | 0.43 | 1 | 1 | 0.50 | 4.93 | 0.89 | 1 | 1 | 0.93 |  |
| 3:7:1 | 42.74 | 0.92 | 1 | 1 | 0.89 | 1.61 | 0.87 | 1 | 1 | 0.78 |  |
